# Fusarochromanone Induces G_1_ Cell Cycle Arrest and Apoptosis in COS7 and HEK293 Cells

**DOI:** 10.1371/journal.pone.0112641

**Published:** 2014-11-10

**Authors:** Ying Gu, Xin Chen, Chaowei Shang, Karnika Singh, Mansoureh Barzegar, Elahe Mahdavian, Brian A. Salvatore, Shanxiang Jiang, Shile Huang

**Affiliations:** 1 Laboratory of Veterinary Pharmacology and Toxicology, College of Veterinary Medicine, Nanjing Agricultural University, Nanjing, Jiangsu Province, P. R. China; 2 Department of Biochemistry and Molecular Biology, Louisiana State University Health Sciences Center, Shreveport, Louisiana, United States of America; 3 Feist-Weiller Cancer Center, Louisiana State University Health Sciences Center, Shreveport, Louisiana, United States of America; 4 Department of Chemistry and Physics, Louisiana State University, Shreveport, Louisiana, United States of America; Roswell Park Cancer Institute, United States of America

## Abstract

Fusarochromanone (FC101), a mycotoxin produced by the fungus *Fusarium equiseti*, is frequently observed in the contaminated grains and feedstuffs, which is toxic to animals and humans. However, the underlying molecular mechanism remains to be defined. In this study, we found that FC101 inhibited cell proliferation and induced cell death in COS7 and HEK293 cells in a concentration-dependent manner. Flow cytometric analysis showed that FC101 induced G_1_ cell cycle arrest and apoptosis in the cells. Concurrently, FC101 downregulated protein expression of cyclin D1, cyclin-dependent kinases (CDK4 and CDK6), and Cdc25A, and upregulated expression of the CDK inhibitors (p21^Cip1^ and p27^Kip1^), resulting in hypophosphorylation of Rb. FC101 also inhibited protein expression of Bcl-2, Bcl-xL, Mcl-1 and survivin, and induced expression of BAD, leading to activation of caspase 3 and cleavage of PARP, indicating caspase-dependent apoptosis. However, Z-VAD-FMK, a pan-caspase inhibitor, only partially prevented FC101-induced cell death, implying that FC101 may induce cell death through both caspase-dependent and -independent mechanisms. Our results support the notion that FC101 executes its toxicity at least by inhibiting cell proliferation and inducing cell death.

## Introduction

Fusarochromanone (FC101) is a mycotoxin produced by *Fusarium equiseti* (*F. equiseti*), a symbiotic fungus, which is frequently found on various decaying cereal plants [1]–[4]. The contamination of FC101 in the feedstuffs was originally discovered to cause avian tibial dyschondroplasia (ATD) in broiler chickens, a disease characterized by bone deformation [2], [5]–[8]. Later, FC101 was also suspected to be associated with etiology of Kashin-Beck disease (a chronic and endemic osteochondropathy) in children in northeastern and southwestern China, as well as in southeastern Siberia and northern Korea [9], [10]. It has been described that FC101 causes ATD probably due to the cytotoxic effect on osteoblasts [11]. In addition, FC101 can inhibit the proliferation of B-16 melanoma cells, MCF-7 breast cancer cells, and normal cardiac fibroblasts [12]. Furthermore, FC101 is cytotoxic and can induce apoptosis in melanoma cells in culture and in mouse xenografts [13]. However, so far, the molecular mechanisms by which FC101 inhibits cell proliferation and induces apoptosis are not well understood.

Cell proliferation is associated with cell cycle progression [14], [15]. Cell cycle is divided into G_0_/G_1_, S, and G_2_/M phases, which is tightly governed by the activity of various cyclin-dependent kinases (CDKs) in mammalian cells [15]. A CDK (catalytic subunit) has to bind to a regulatory subunit, cyclin, to become active [15]. Also, Wee1 phosphorylates specific residues (Tyr15 and Thr14) of CDKs, inhibiting CDKs, which is counteracted by Cdc25 through dephosphorylation [15]. However, cyclin activating kinase (CAK) phosphorylates CDKs (Thr161), activating CDKs [15]. Furthermore, p21^Cip1^ and p27^Kip1^, two universal CDK inhibitors, can bind a CDK, inhibiting the CDK activity and the cell cycle progression [14], [16]. Cyclin D-CDK4/6 and cyclin E-CDK2 complexes control G_1_ cell cycle progression, whereas cyclin A-CDK2 and cyclin B-CDK1 regulate S and G_2_/M cell cycle progression, respectively [15]. Therefore, perturbing expression of CDKs and/or the regulatory proteins, such as cyclins, Cdc25 and CDK inhibitors, may affect cell cycle progression.

Apoptosis is a type of programmed cell death and occurs actively in multicellular organisms under physiological and pathological conditions [17]. Under physiological condition, if cells are unwanted any more, they will commit suicide by activating an intracellular death program to ensure the proper tissue/organ homeostasis; under pathological conditions (such as viral infection, UV or ionizing irradiation, chemotherapeutic agents, etc.), if cells are damaged too severely to repair, they will also undergo apoptosis through intrinsic/extrinsic pathways to maintain the genomic stability [17]. Apoptosis can be triggered through caspase-dependent/independent mechanisms [17]. Among many proteins, the Bcl-2 family members are key players in the regulation of apoptosis [18]. The Bcl-2 family consists of anti-apoptotic (e.g. Bcl-2, Bcl-xL, and Mcl-1) and pro-apoptotic proteins (e.g. BAD, BAK, and BAX) that work together and with other proteins to maintain a dynamic balance between the cell survival and the cell death [18].

This study was set to investigate the molecular mechanisms by which FC101 inhibits cell proliferation and induces cell death. We found that FC101 inhibited cell proliferation by arresting cells in G_1_ phase of the cell cycle, due to altered expression of the cell cycle related proteins in COS7 and HEK293 cells. Furthermore, FC101 induced caspase-dependent apoptosis by disrupting the balance between pro-apoptotic and anti-apoptotic proteins.

## Materials and Methods

### Materials

Fusarochromanone (FC101) is also known as NSC627608 (IUPAC Name: 5-amino-6-[(3R)-3-amino-4- hydroxybutanoyl]-2,2-dimethyl-3H-chromen-4-one; molecular formula: C_15_H_20_N_2_O_4_; molecular weight: 292.3). FC101 was isolated and purified (purity >97%, by NMR) from rice cultures of the fungus *F. equiseti*, and was converted to the stable and water-soluble phosphate-salt form, as described [13]. The phosphate-salt form of FC101 was then dissolved in Milli-Q water and filtered through a 0.2 µm syringe filter to prepare a sterile stock solution (5 mM), aliquoted and stored at −20°C. Dulbecco’s modified Eagle’s medium (DMEM), MEM non-essential amino acids and 0.05% trypsin-EDTA were obtained from Mediatech (Herndon, VA). Fetal bovine serum (FBS) was from Atlanta Biologicals (Lawrenceville, GA). Enhanced chemiluminescence solution was from Perkin-Elmer Life Science (Boston, MA). CellTiter 96 AQueous One Solution Cell Proliferation Assay kit was from Promega (Madison, WI). Annexin V-FITC Apoptosis Detection Kit I was purchased from BD Biosciences (San Jose, CA). The following antibodies were used: cyclin A, cyclin B1, cyclin D1, cyclin E, p21^Cip1^, p27^Kip1^, Cdc25A, Cdc25B, Cdc25C, CDK1, CDK2, CDK4, Rb, p-Rb (S807/811), BAD, BAX, BAK, Bcl-2, Bcl-xL, Mcl-1, survivin, caspase 3, PARP (Santa Cruz Biotechnology, Santa Cruz, CA), cleaved caspase 3, cleaved PARP (Cell Signaling, Beverly, MA), β-tubulin (Sigma, St Louis, MO), goat anti-mouse IgG-horseradish peroxidase, and goat anti-rabbit IgG-horseradish peroxidase (Pierce, Rockford, IL).

### Cell lines and cultures

Both African green monkey kidney fibroblast-like cells (COS7) and human embryonic kidney 293 cells (HEK 293) were purchased from American Type Culture Collection (Manassas, VA), and grown in antibiotic-free DMEM supplemented with 10% FBS and 1% MEM non-essential amino acids. All cells were cultured in a humid incubator (37°C and 5% CO_2_).

### Cell morphological analysis and cell proliferation assay

COS7 and HEK 293 cells were seeded at a density of 2×10^4^ cells per well in 6-well plates. The next day, FC101 (0–5 µM) was added. After incubation for 4–6 days until the control cells were nearly 100% confluent, images were taken with an Olympus inverted phase-contrast microscope equipped with the Quick Imaging system. All cells were then trypsinized and enumerated using a Z1 Coulter Counter (Beckman Coulter, Fullerton, CA). Cells treated with vehicle alone (phosphate buffered saline, PBS) were used as a control.

### One solution assay

Cell proliferation was also evaluated by one solution assay, using CellTiter 96 AQueous One Solution Cell Proliferation Assay kit (Promega), according the manufacturer’s instructions. Briefly, cells suspended in the growth medium were seeded in a 96-well plate at a density of 1×10^4^ cells per well (in triplicates). The next day, FC101 (0–5 µM) was added. After incubation for 24 h, each well was added 20 µl of one solution reagent and incubated for 1 h. Cell proliferation was determined by measuring the optical density (OD) at 490 nm using a Wallac 1420 Multilabel Counter (PerkinElmer Life Sciences, Wellesley, MA). Cells treated with vehicle alone (PBS) served as a control.

### Cell cycle analysis

Cells were seeded in 100-mm dishes at a density of 5×10^5^ cells per dish in DMEM supplemented with 10% FBS and grown overnight at 37°C in a humidified incubator with 5% CO_2_. The next day, cells were then treated with FC101 at 0–5 µM for 24 h or at 1 µM for 0–72 h. Subsequently, the cells were briefly washed with PBS and trypsinized. Cell suspensions were centrifuged at 1,000 rpm for 5 min, and the pellets were fixed with pre-chilled (−20°C) absolute ethanol and stained with the Cellular DNA Flow Cytometric Analysis Kit (Roche Diagnostics Corp., Indianapolis, IN). Percentages of cells within each of the cell cycle compartments (G_0_/G_1_, S, or G_2_/M) were determined using a FACSCalibur flow cytometer (Becton Dickinson, San Jose, CA) and ModFit LT analyzing software (Verity Software House, Topsham, ME). Cells treated with vehicle alone (PBS) served as a control. Also, the confluence of the control cells was kept <80%, to minimize a possible effect of the cell-cell contact inhibition on cell cycle progression.

### Trypan blue exclusion assay

Cell viability was evaluated by the trypan blue exclusion assay. Briefly, cells were seeded in 60-mm dishes at a density of 3×10^5^ cells per dish in the growth medium and grown overnight at 37°C in a humidified incubator with 5% CO_2_. Following treatment with FC101 (0–1 µM) for 24–72 h, the cells (including floating and attached cells) were harvested, pelleted, and resuspended in 1 ml of PBS. Then, 1 part of cell suspension was incubated with 1 part of 0.4% trypan blue solution (Sigma) for 3 min at room temperature. Finally, 10 µl of the trypan blue/cell mixture was applied to a hemacytometer, and the unstained (viable) and stained (nonviable) cells were counted separately under a microscope. For each treatment, at least 300 cells (total) were counted, and the percentage of the stained cells will be calculated.

### Apoptosis assay

Cells were seeded in 60-mm dishes at a density of 3×10^5^ cells per dish in the growth medium and grown overnight at 37°C in a humidified incubator with 5% CO_2_. Following treatment with FC101 (0–5 µM) for 72 h, the cells (including floating and attached cells) were harvested, followed by apoptosis assay using the Annexin V-FITC Apoptosis Detection Kit I (BD Biosciences, San Jose, CA), according the manufacturer’s instructions. Flow cytometry was performed using a FACSCalibur flow cytometer (Becton Dickinson). Cells treated with vehicle alone (PBS) served as a control.

### Caspase-3/7 assay

Cells were seeded in 96-well black plates at a density of 1×10^4^ cells/well under standard culture conditions and kept overnight at 37°C humidified incubator with 5% CO_2_. The next day, the cells were preincubated without or with Z-VAD-FMK (10 µM) for 1 h and then treated without or with FC101 (0–5 µM) for 24 h. Caspase-3/7 activity was measured using the SensoLyte Homogeneous AMC Caspase3/7 Assay kit (AnaSpec, Fremont, CA) according to the manufacturer’s protocol. In brief, 50 µl of caspase-3/7 substrate solution was added into each well. After incubation for 9 h at room temperature, the fluorescence intensity was measured by excitation at 355 nm and emission at 460 nm using a Wallac 1420 Multilabel Counter (PerkinElmer Life Sciences, Wellesley, MA).

### Western blot analysis

Cells were seeded in 6-well plates under standard culture conditions and kept overnight at 37°C humidified incubator with 5% CO_2_. The cells were treated with FC101 (0–5 µM) for 24 h, and then briefly washed with cold PBS. On ice, cells were lysed in RIPA buffer, containing 50 mM Tris, pH 7.2; 150 mM NaCl; 1% sodium deoxycholate; 0.1% sodium dodecyl sulfate; 1% Triton-X 100; 10 mM NaF; 1 mM Na_3_VO_4_; protease inhibitor cocktail (1∶1000, Sigma). Lysates were sonicated for 10 s and centrifuged at 14,000 rpm for 10 min at 4°C. Protein concentration was determined by bicinchoninic acid assay with bovine serum albumin as a standard (Pierce). Equivalent amounts of protein was separated on 6–15% sodium dodecyl sulfate-polyacrylamide gels and transferred to polyvinylidene difluoride membranes (Millipore, Bedford, MA). Membranes were incubated with PBS containing 0.05% Tween 20 and 5% non-fat dry milk to block non-specific binding, and then with the primary antibodies, followed by incubation with appropriate secondary antibodies conjugated to horseradish peroxidase. Immunoreactive bands were visualized by using Renaissance chemiluminescence reagent (Perkin-Elmer Life Science).

### Statistical analysis

Results were expressed as mean values ± SE (standard error). Statistical analysis was performed by Student’s *t*-test (STATISTICA, Statsoft Inc, Tulsa, OK). A level of *P*<0.05 was considered to be significant.

## Results

### FC101 inhibits cell proliferation and reduces cell viability

**Figure 1:**
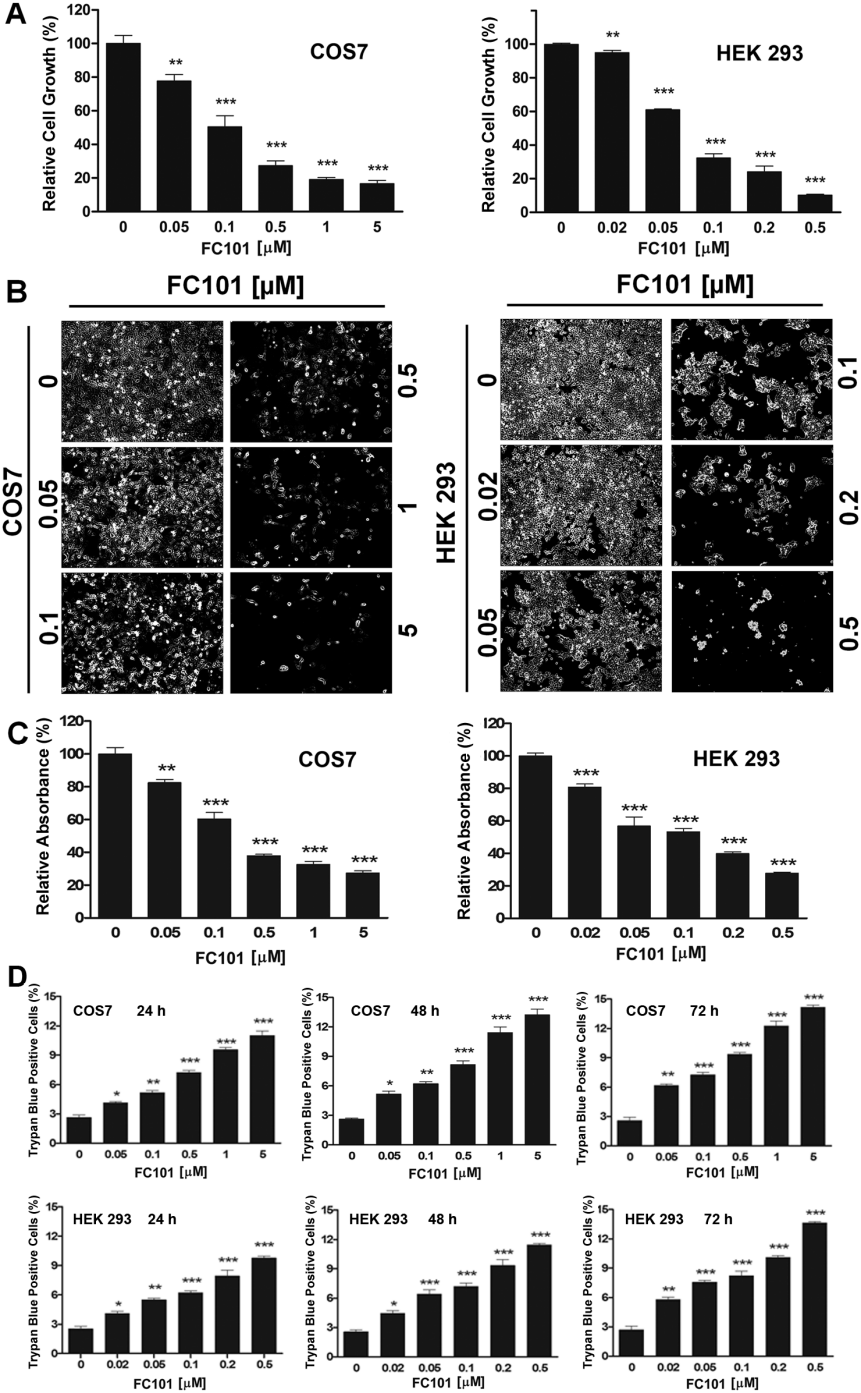
FC101 inhibits cell proliferation and reduces cell viability. COS7 and HEK 293 cells were treated with FC101 (0–5 µM) for 6 days (for COS7) or 4 days (for HEK 293) (A, B), or 48 h (C, D), followed by cell number counting (A), morphological analysis (B), one solution assay (C), and trypan blue exclusion assay (D). For (A), (C), and (D), data represents mean ± SE (n = 6). *P<0.05, **P<0.01, ***P<0.001, difference with the control group (FC101 = 0 µM).

To study the toxic effects of FC101 on cells, first of all, we tested whether FC101 affects cell proliferation and cell viability. For this, COS7 were treated with FC101 for 6 days at concentrations of 0–5 µM. We found that FC101 inhibited cell proliferation in a concentration-dependent manner (Fig. 1A and 1B). Similar results were observed in HEK 293 cells (Fig. 1A, 1B, and Fig. S1A). The IC_50_ of FC101 was ∼0.1 µM for COS7 and ∼0.07 µM for HEK 293 cells, respectively. Besides, when the cells were exposed to the compound for 24–72 h, one solution assay also revealed a similar growth inhibitory pattern (Fig. 1C and Fig. S1B). Moreover, FC101 also reduced cell viability in a concentration- and time-dependent manner, as judged by trypan blue exclusion assay (Fig. 1D). Therefore, the results suggest that FC101 inhibits cell proliferation and induces cell death in COS7 and HEK 293 cells.

### FC101 arrests cells at G_0_/G_1_ phase of the cell cycle

**Figure 2:**
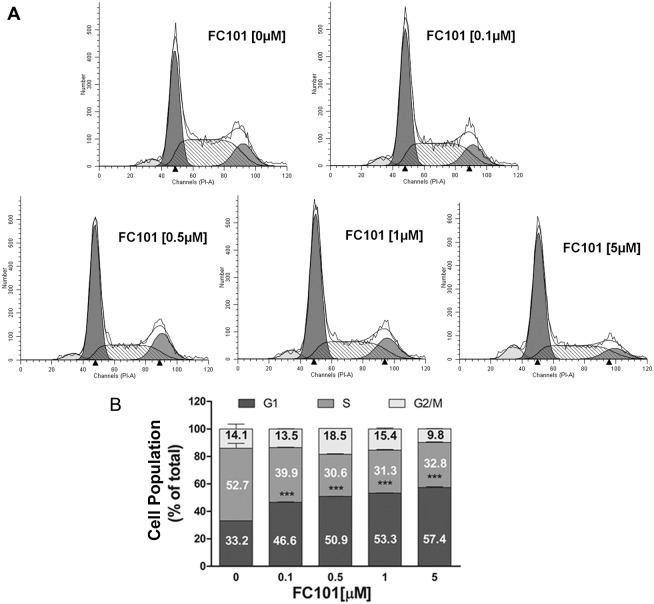
FC101 arrests cells at G_0_/G_1_ phase of the cell cycle. COS7 cells were treated with FC101 (0–5 µM) for 24 h. The cells were then harvested and processed for cell cycle analysis using Cellular DNA Flow Cytometric Analysis Kit and flow cytometry. (A) Histograms from a representative experiment show the effect of FC101 on cell cycle profile in COS7 cells. (B) Bar graphs show the effect of FC101 on the distribution (%) of COS7 cells in the G_0_/G_1_, S and G_2_/M phases of the cell cycle. Data represents mean ± SE (n = 3). *P<0.05, **P<0.01, ***P<0.001, difference with the control group (FC101 = 0 µM).

To understand how FC101 inhibits cell proliferation, cell cycle analysis was performed. Since COS7 and HEK 293 have a doubling time of approximately 18 h and 10 h, respectively (http://www.synvolux.com/dna-transfection-reagent/cell-doubling-time-in-transfection), the cells were treated with FC101 for 24 h, followed by cell cycle analysis. As shown in Fig. 2, treatment with FC101 for 24 h induced cell cycle arrest at G_0_/G_1_ phase in COS7 cells in a concentration-dependent manner. FC101 treatment significantly increased the proportion of cells in the G_0_/G_1_ phase from 33.2% to 57.4%. In addition, treatment with FC101 (1 µM) for up to 72 h also induced a time-dependent cell cycle arrest at G_0_/G_1_ phase in COS7 cells (data not shown). Similar data were observed in HEK293 cells (Fig. S2). Our findings indicate that FC101 inhibits cell proliferation by arresting cells at G_0_/G_1_ phase of the cell cycle.

### FC101 downregulates expression of cyclin D1, CDK4/6 and Cdc25A and upregulates expression of CDK inhibitors (p21^Cip1^ and p27^Kip1^), leading to dephosphorylation of Rb

**Figure 3:**
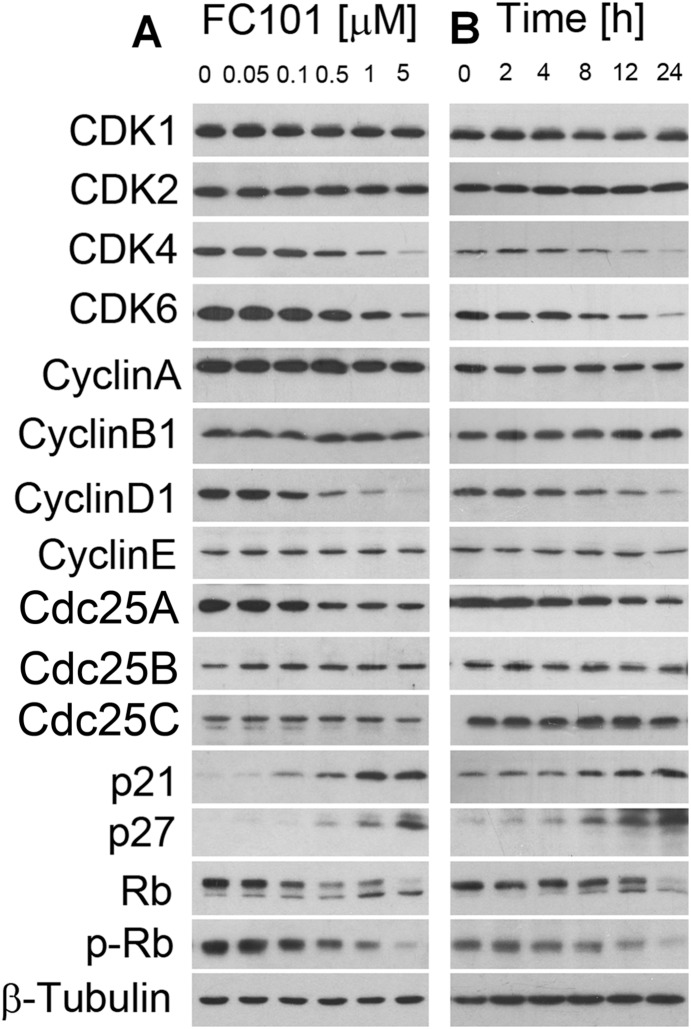
FC101 downregulates protein expression of cyclin D1, Cdc25A, CDK4/6 and upregulates expression of p21^Cip1^ and p27^Kip1^, leading to hypophosphorylation of Rb. COS7 cells were treated with FC101 for 24 h at indicated concentrations (A), or treated with FC101 at 1 µM for indicated time (B), followed by Western blotting with indicated antibodies. β-Tubulin was used for loading control. Representative blots are shown. Similar results were observed in at least 3 independent experiments.

To further unravel the mechanism by which FC101 arrests cells at G_0_/G_1_ phase of the cell cycle, we examined expression of CDKs and related regulatory proteins, including cyclins, Cdc25 and CDK inhibitors. As shown in Fig. 3A, treatment of COS7 with FC101 for 24 h downregulated cellular protein expression of cyclin D1, CDK4, CDK6, and Cdc25A, and upregulated protein expression of two CDK inhibitors, p21^Cip1^ and p27^Kip1^, in a concentration-dependent manner. As Rb, one of the most important G_1_-cyclin/CDK substrates, functions as a regulator of cell cycle progression in the late G_1_ phase [19], we further investigated the effect of FC101 on Rb phosphorylation. As shown in Fig. 3, Rb was expressed as a 110-kDa band in control cells. After FC101 treatment at 1 µM for 24 h, a lower band, which migrates rapidly and represents the dephosphorylated protein, was observed (Fig. 3), indicating that FC101 inhibited phosphorylation of Rb. This was further verified by using the antibodies to phospho-Rb (S807/811) (Fig. 3A). In addition, FC101 (1 µM) also inhibited expression of cyclin D1, CDK4/6 and Cdc25A, as well as phosphorylation of Rb in a time-dependent manner (Fig. 3B). Similar results were also observed in HEK 293 cells (Fig. S3). Our results suggest that FC101 arrested cells in G_0_/G_1_ phase of the cell cycle by downregulating expression of cyclin D1, CDK4/6 and Cdc25A and upregulating expression of CDK inhibitors (p21^Cip1^ and p27^Kip1^), leading to dephosphorylation of Rb.

### FC101 induces apoptosis by downregulating expression of anti-apoptotic proteins (Mcl-1, Bcl-2, Bcl-xL and survivin) and upregulating expression of pro-apoptotic protein BAD

**Figure 4:**
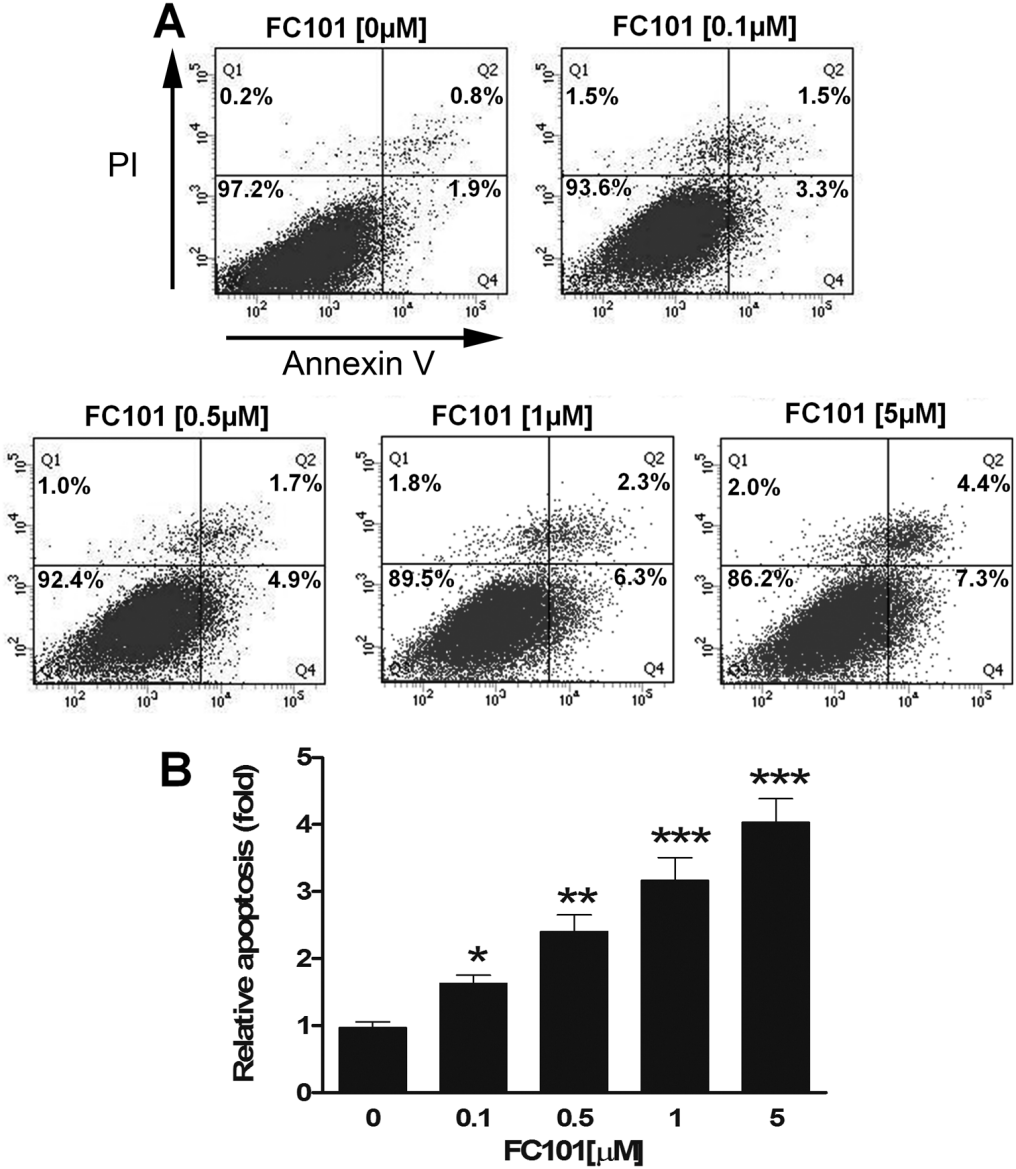
FC101 induces apoptosis. COS7 cells were treated with FC101 (0–5 µM) for 72 h. The cells were harvested and processed for apoptosis assay using the Annexin V-FITC Apoptosis Detection Kit. The cell distribution was analyzed by flow cytometry. (A) Histograms from a representative experiment show the apoptotic effect of FC101 on COS7 cells. The percentages of necrotic, late apoptotic, viable, and early apoptotic cells are displayed in Q1, Q2, Q3 and Q4, respectively. (B) Bar graphs show that FC101 induced apoptosis of COS7 cells in a concentration-dependent manner. Quantitative results (Q2+Q4) are displayed as fold change compared with control. Data represents mean ± SE (n = 3). *P<0.05, **P<0.01, ***P<0.001, difference with the control group (FC101 = 0 µM).

According to our trypan blue exclusion assay (Fig. 1D), treatment with FC101 for 72 h was able to induce concentration-dependent cell death in COS7 and HEK293 cells. To determine whether FC101 induces apoptotic cell death in the cells, we performed Annexin V-FITC/PI staining, a method that is frequently used to detect apoptosis [20]. As shown in Fig. 4, treatment with FC101 for 72 h induced apoptosis in COS7 cells in a concentration-dependent manner. FC101 at 0.1–5 µM increased the proportion (Q2+Q4) of cells positive for Annexin-V/PI by approximately 1.8–4.3 fold, compared to the control.

**Figure 5:**
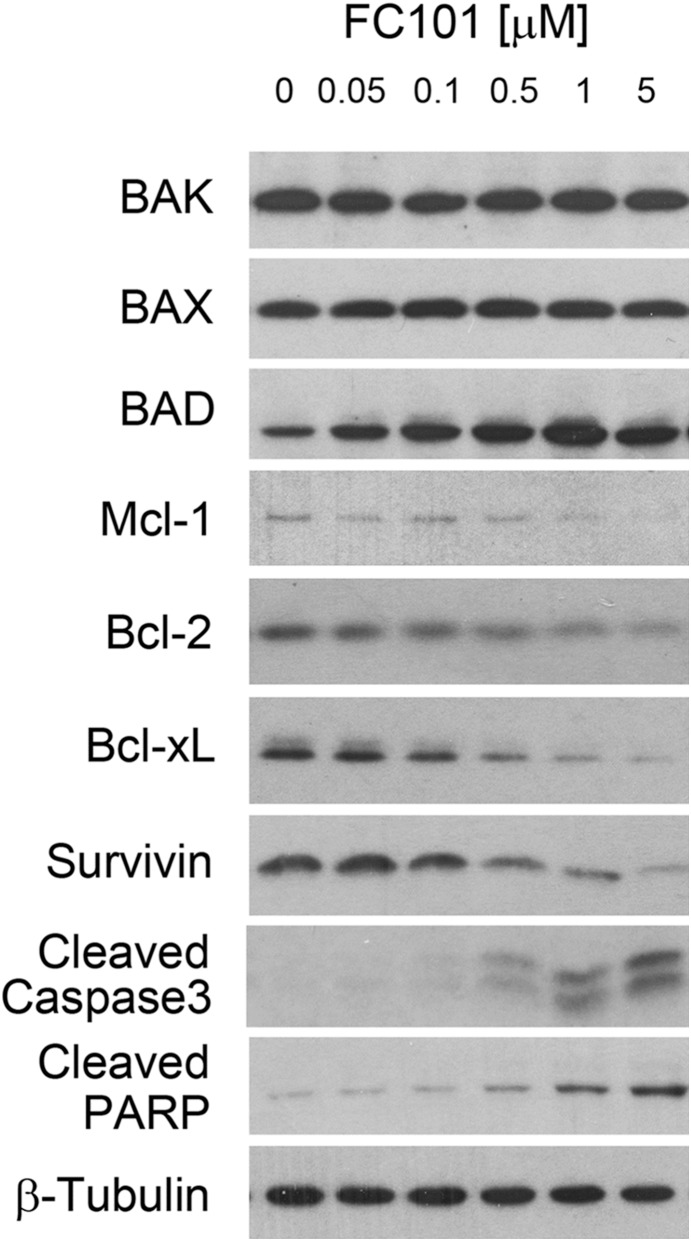
FC101 downregulates expression of the anti-apoptotic proteins (Bcl-2, Mcl-1, Bcl-xL and survivin) and increases expression of the pro-apoptotic protein BAD, increasing cleavages of caspase 3 and PARP. COS7 cells were treated with FC101 for 24 h at indicated concentrations, followed by Western blotting with indicated antibodies. β-Tubulin served as a loading control. Representative blots are shown. Similar results were observed in at least 3 independent experiments.

In order to elucidate how FC101 induces apoptosis, we next examined whether FC101 alters expression of pro-apoptotic and anti-apoptotic proteins in the cells. As shown in Fig. 5, treatment with FC101 for 24 h markedly downregulated expression levels of anti-apoptotic proteins (Mcl-1, Bcl-2, Bcl-xL and survivin) and meanwhile upregulated pro-apoptotic proteins (BAD) in a concentration-dependent manner. It appeared that after 24 h treatment, FC101 did not obviously alter expression of two other pro-apoptotic proteins BAK and BAX. Furthermore, we observed that FC101 concentration-dependently induced PARP cleavage (Fig. 5 and Fig. S4), a hallmark of caspase-dependent apoptosis. This was consistent with the data that FC101 increased cleavage of caspase 3, indicating activation of caspase 3 (Fig. 5 and Fig. S4).

### FC101-induced cell death is partially caspase-dependent

**Figure 6:**
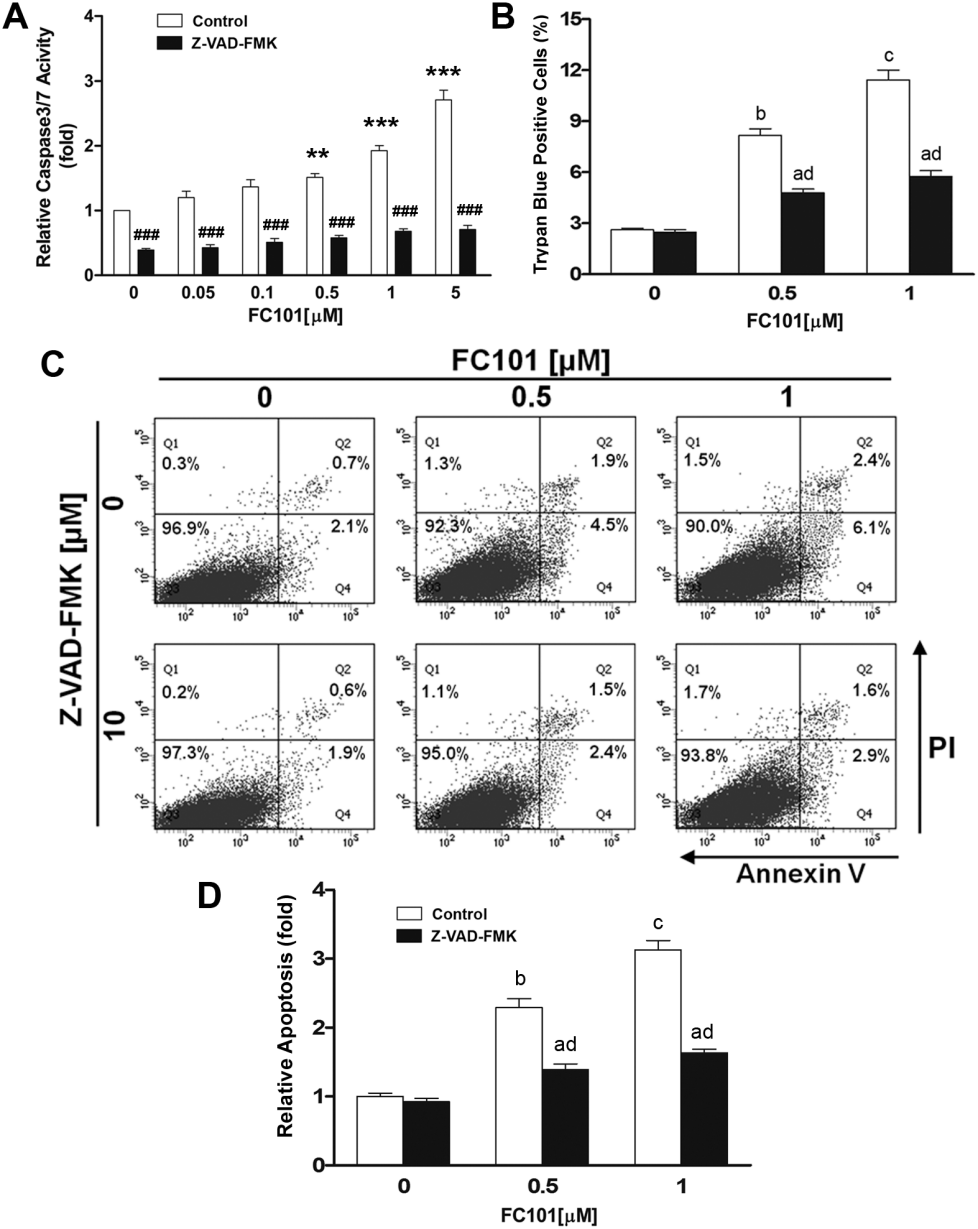
FC101 induces caspase-dependent apoptosis. (A–D) COS7 cells, pretreated with or without Z-VAD-FMK (10 µM) for 1 h, were incubated with FC101 at indicated concentrations for 24 h, followed by caspase 3/7 activity assay (A), for 48 h, followed by trypan blue exclusion assay (B), or for 72 h, followed by Annexin V-FITC/PI staining and flow cytometry (C, D). (C) Histograms from a representative experiment show the effect of Z-VAD-FMK on FC101-induced apoptosis of COS7 cells. The percentages of necrotic, late apoptotic, viable, and early apoptotic cells are displayed in Q1, Q2, Q3 and Q4, respectively. (D) Bar graphs show that Z-VAD-FMK partially prevented FC101-induced apoptosis of COS7 cells. Quantitative results (Q2+Q4) were displayed as fold change compared with control. For (A), (B), and (D), data represents mean ± SE (n = 3). ^a^ *P*<0.05, ^b^ *P*<0.01, ^c^ *P*<0.001, difference with the control group (FC101 = 0 µM). ^d^ *P*<0.01, difference with Z-VAD-FMK group.

To further explore whether FC101 induces cell death is fully through caspase-dependent mechanism, Z-VAD-FMK, a pan-caspase inhibitor, was employed. In line with the above Western blotting result (Fig. 5), treatment with FC101 (0.5–5 µM) for 24 h significantly increased caspase 3/7 activities (Fig. 6A), as detected using the SensoLyte Homogeneous AMC Caspase 3/7 Assay kit. Pretreatment of cells with Z-VAD-FMK (10 µM) for 1 h almost completely blocked FC101-induced activation of caspase 3/7 (Fig. 6A). However, pretreatment with Z-VAD-FMK only partially prevented FC101-induced cell death, as detected by the trypan blue exclusion assay (Fig. 6B). This was further verified by Annexin V-PI staining (Fig. 6C and 6D). Therefore, our results suggest that FC101 may induce cell death through caspase-dependent and -independent mechanisms.

## Discussion

FC101 is a toxic fungal metabolite mainly produced by *F. equiseti* [13], which has been observed frequently in human food and animal feed [1]–[4], [21]. FC101 was originally discovered to cause tibial dyschondroplasia in rapidly growing chickens [2], [5]–[8]. Also, FC101-contaminated environment or food chain has been speculated to be an etiopathogenic factor of Kashin-Beck disease in children [9], [10]. Although the mechanisms of the toxic actions in animals and humans remain to be determined, studies have shown that FC101 can inhibit cell proliferation and induce cell death in lymphocytes, A375, CMC9209 and B16 melanoma cells, MCF-7 breast cancer cells, as well as cardiac fibroblasts [11]–[13]. Nevertheless, the precise molecular mechanisms by which FC101 inhibits cell proliferation and induces cell death are not well understood. Here, for the first time, we present evidence that FC101 inhibited cell proliferation in COS7 and HEK293 cells, by downregulating protein expression of cyclin D1, cyclin-dependent kinases (CDK4 and CDK6), and Cdc25A, and upregulating expression of the CDK inhibitors (p21^Cip1^ and p27^Kip1^), leading to hypophosphorylation of Rb and cell cycle arrest at G_1_ phase. Furthermore, FC101 induced apoptosis by inhibiting protein expression of Bcl-2, Bcl-xL, Mcl-1 and survivin, and inducing expression of BAD, resulting in caspase-dependent apoptosis.

As FC101 has been detected in the feed samples at concentrations of 4–59 µg/kg, which caused tibial dyschondroplasia in chickens, and also *F. equiseti* strains can produce FC101 at 57–1,435 mg/kg in cereals in the laboratory [2], 0–5 µM (corresponding to 0–1,462 µg/L) of FC101 was selected in this study. For instance, we found that FC101 inhibited cell proliferation by arresting the cells in the G_0_/G_1_ phase of the cell cycle. This is different from the findings in other mycotoxins, such as zearalenone, nivalenol, deoxynivalenol, and fumonisin B1 [22], [23], but is in agreement with the observations in T-2 toxin and ochratoxin A [24], [25]. For example, it has been described that zearalenone, a non-steroidal oestrogenic mycotoxin produced by some *Fusarium* and *Gibberella* species, increases cell population in the G_2_/M phase of the cell cycle in Vero, Caco-2 and DOK cells [22]; T-2 toxin, a member of the trichothecene mycotoxin family produced by the *Fusarium* fungi, inhibits cell cycle progression by arresting cells at G_0_/G_1_ phase in murine embryonic stem cells [24], and ochratoxin A, a toxin produced by *Aspergillus ochraceus*, *Aspergillus carbonarius* and *Penicillium verrucosum*, induces G_0_/G_1_ phase arrest in human peripheral blood mononuclear cells [25]. Of note, ochratoxin A has also been reported to induce G_2_/M phase arrest in human gastric endothelial cells [26], suggesting that the effect of ochratoxin A on the cell cycle profile is cell-type dependent. It is unknown whether FC101, like ochratoxin A, can also induce G_2_/M or S phase arrest in other cells. Further research using more cell lines may address this issue.

In eukaryotes, cell cycle progression is regulated by a series of cyclins/CDK, CDK inhibitors and Cdc25 phosphatase [15], [27]. Early G_1_ transition is mainly regulated by cyclin D1 complexed with CDK4 and/or CDK6, whereas late G_1_-S and early S-phase transitions are regulated by cyclin E coupled with CDK2 [15], [28]. Among the three Cdc25 isoforms (Cdc25A/B/C) present in mammalian cells, which activate CDKs at different phases of the cell cycle through dephosphorylation of the CDKs, Cdc25A is the only member required for the control of G_1_/S CDKs’ activities [29], [30]. To investigate how FC101 arrests the cells in G_0_/G_1_ phase, we examined the effects of FC101 on the expression of cell cycle regulatory proteins. Our Western blot data (Fig. 3) indicated that FC101 downregulated protein expression of cyclin D1 and its enzymatic counterparts CDK4/CDK6, as well as Cdc25A. In addition, FC101 potently induced expression of two CDK inhibitors, p21^Cip1^ and p27^Kip1^, which can bind and inhibit G_1_ CDKs [16], [31]. As a result, the phosphorylation of Rb was inhibited, leading to G_1_ arrest. Taken together, our results indicate that FC101-induced G_1_ cell cycle arrest is a consequence of the inhibition of G_1_-CDKs, related to downregulated expression of cyclin D1, CDK4/6, Cdc25A and upregulated expression of CDK inhibitors (p21^Cip1^ and p27^Kip1^).

Apoptosis is a complex process that is tightly regulated by the balance of pro-apoptotic proteins (e.g. BAX, BAD and BAK) and anti-apoptotic proteins (e.g. Bcl-xL, Bcl-2, and Mcl-1) [17], [32], [33]. In the present study, we found that FC101 induced apoptosis by reducing expression of the anti-apoptotic proteins including Bcl-xL, Bcl-2, Mcl-1 and survivin, and in the meantime increasing expression of the pro-apoptotic protein BAD (Fig. 5). This might result in a dominance of pro-apoptotic proteins over anti-apoptotic proteins in the cells, leading to apoptotic cell death.

Apoptosis can occur through caspase-dependent and -independent mechanisms [34], [35]. We noticed that FC101 induced cleavages of caspase-3 and PARP (Fig. 5), suggesting a caspase-dependent apoptotic mechanism involved. This is in line with the previous observations that FC101 induces activation of caspase 3 in CMC9209 melanoma xenografts in SCID mice [13], and increases cleavage of PARP in A172 and U251 glioblastoma cells [36]. To confirm the role of caspase cascade in FC101-induced cell death, Z-VAD-FMK, a pan-caspase inhibitor, was used. Interestingly, Z-VAD-FMK (10 µM) almost completely blocked FC101-induced caspase-3/7 activity, but only partially prevented FC101-induced cell death in COS7 and HEK293 cells. Our data imply that FC101 induced cell death probably through both caspase-dependent and -independent mechanisms. This is indeed supported by our flow cytometric results that FC101 did increase necrosis by 5–10 fold (see Q1, control versus FC101, Fig. 4A). More studies are required to unveil how the necrosis (or necroptosis) is induced. It would be also interesting to determine whether FC101 can induce autophagy, which may contribute to caspase-independent cell death as well.

In summary, the present study has demonstrated that FC101 inhibited cell proliferation and induced apoptosis in COS7 and HEK293 cells. Mechanistically, FC101 inhibited cell proliferation by slowing down cell cycle progression from G_1_ to S phase. This was related to downregulation of cyclin D1, CDKs (CDK4 and CDK6) and Cdc25A, and upregulation of CDK inhibitors p21^Cip1^ and p27^Kip1^, resulting in hypophosphorylation of Rb. FC101 induction of apoptosis was associated with downregulation of the anti-apoptotic proteins (Bcl-2, Bcl-xL, Mcl-1 and survivin) and upregulation of the pro-apoptotic protein (BAD). Furthermore, FC101 induced cell death via caspase-dependent and -independent mechanisms.

## Supporting Information

Figure S1 **FC101 inhibits cell proliferation.** (A) HEK 293 cells were treated with FC101 (0–5 µM) for 4 days, followed by taking images under a phase-contrast microscope equipped a digital camera. (B) COS7 and HEK 293 cells were treated with FC101 at indicated concentrations for 24–72 h, followed by one solution assay. Results represent mean ± SE (n = 6). *P<0.05, **P<0.01, ***P<0.001, difference with the control group (FC101 = 0 µM).
(TIF)

Figure S2 **FC101 induces G_0_/G_1_ cell cycle arrest in HEK293 cells.** HEK293 cells were treated with FC101 (0.5 µM) for indicated time. The cells were then harvested and processed for cell cycle analysis using Cellular DNA Flow Cytometric Analysis Kit and flow cytometry. Results are presented as means ± SE (n = 3). *P<0.05, **P<0.01.
(TIF)

Figure S3 **FC101 downregulates expression of cyclin D1, Cdc25A, CDK4/6 and upregulates expression of p21^Cip1^ and p27^Kip1^, leading to hypophosphorylation of Rb in HEK293 cells.** HEK 293 cells were treated with FC101 for 24 h at indicated concentrations (A), or treated with FC101 at 1 µM for indicated time (B), followed by Western blotting with indicated antibodies. β-Tubulin served as a loading control.
(TIF)

Figure S4 **FC101 induces cleavages of caspase 3 and PARP.** COS7 cells were treated with FC101 for 24 h at indicated concentrations, followed by Western blotting with indicated antibodies. β-Tubulin served as a loading control. Representative blots are shown. Similar results were observed in at least 3 independent experiments.
(TIF)
