# Supplementary figures and images for: Fusarochromanone Induces G1 Cell Cycle Arrest and Apoptosis in COS7 and HEK293 Cells
Source: PLoS One. 2014 Nov 10;9(11):e112641. doi: 10.1371/journal.pone.0112641 (PMC4226581; doi:10.1371/journal.pone.0112641)

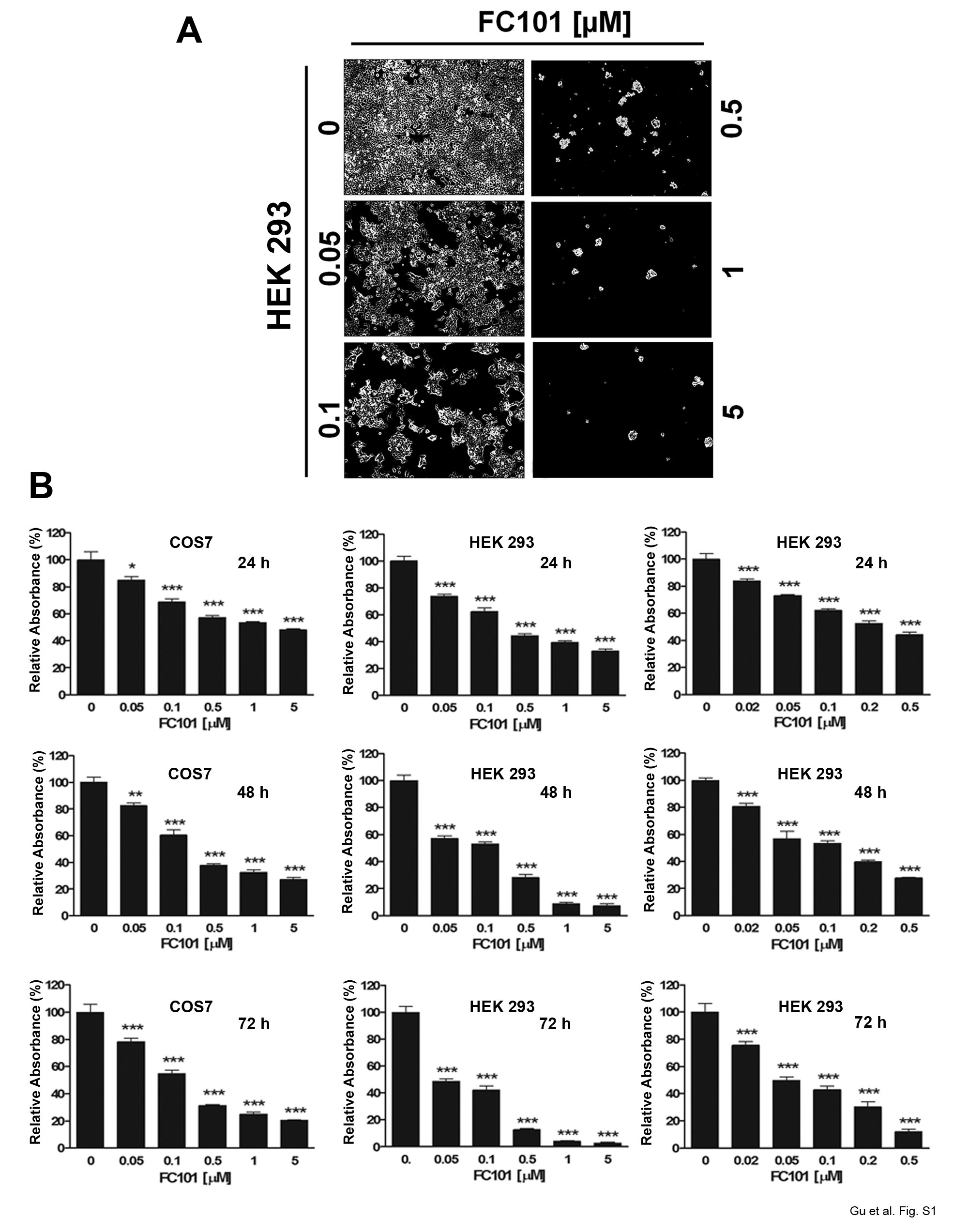

Supplement: Figure S1 — FC101 inhibits cell proliferation. (A) HEK 293 cells were treated with FC101 (0–5 µM) for 4 days, followed by taking images under a phase-contrast microscope equipped a digital camera. (B) COS7 and HEK 293 cells were treated with FC101 at indicated concentrations for 24–72 h, followed by one solution assay. Results represent mean ± SE (n = 6). *P<0.05, **P<0.01, ***P<0.001, difference with the control group (FC101 = 0 µM). (TIF) [file pone.0112641.s001.tif]

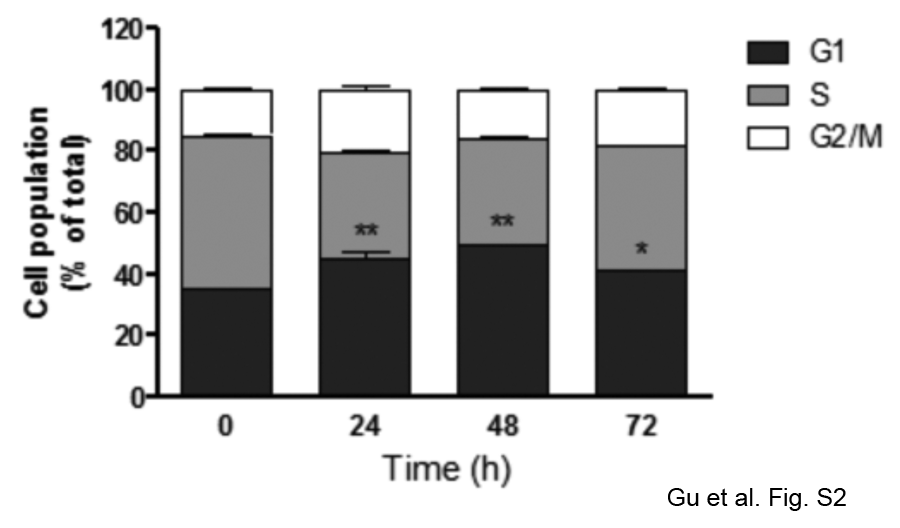

Supplement: Figure S2 — FC101 induces G0/G1 cell cycle arrest in HEK293 cells. HEK293 cells were treated with FC101 (0.5 µM) for indicated time. The cells were then harvested and processed for cell cycle analysis using Cellular DNA Flow Cytometric Analysis Kit and flow cytometry. Results are presented as means ± SE (n = 3). *P<0.05, **P<0.01. (TIF) [file pone.0112641.s002.tif]

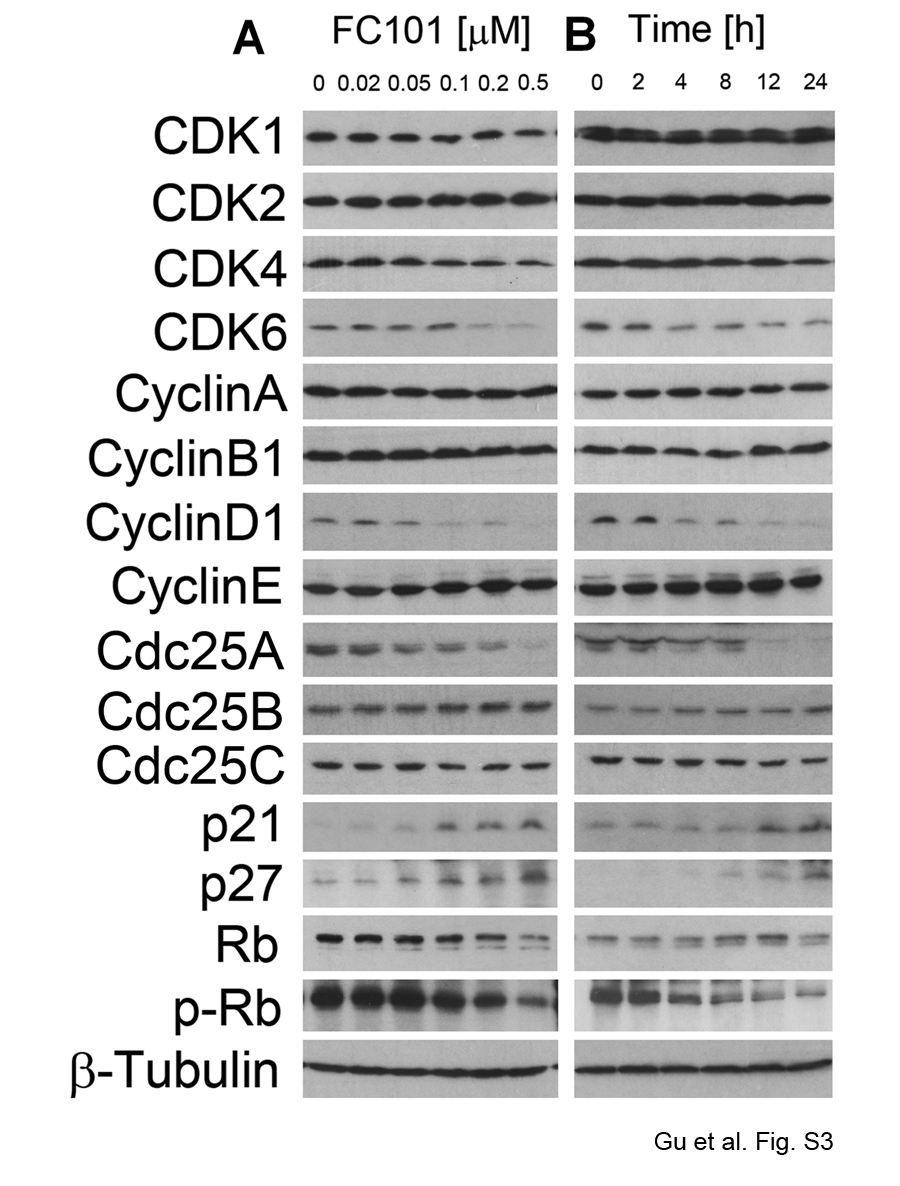

Supplement: Figure S3 — FC101 downregulates expression of cyclin D1, Cdc25A, CDK4/6 and upregulates expression of p21Cip1 and p27Kip1, leading to hypophosphorylation of Rb in HEK293 cells. HEK 293 cells were treated with FC101 for 24 h at indicated concentrations (A), or treated with FC101 at 1 µM for indicated time (B), followed by Western blotting with indicated antibodies. β-Tubulin served as a loading control. (TIF) [file pone.0112641.s003.tif]

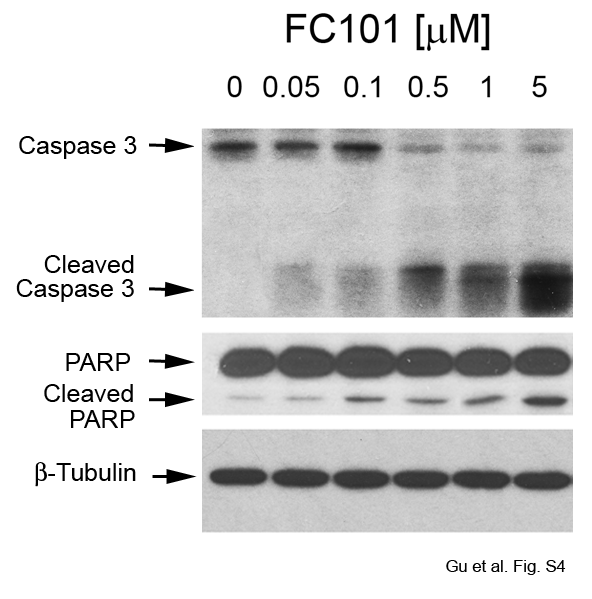

Supplement: Figure S4 — FC101 induces cleavages of caspase 3 and PARP. COS7 cells were treated with FC101 for 24 h at indicated concentrations, followed by Western blotting with indicated antibodies. β-Tubulin served as a loading control. Representative blots are shown. Similar results were observed in at least 3 independent experiments. (TIF) [file pone.0112641.s004.tif]
